# Association between albumin-to-alkaline phosphatase ratio and a 3-month unfavorable outcome in patients with acute ischemic stroke

**DOI:** 10.3389/fnut.2025.1537954

**Published:** 2025-04-03

**Authors:** Renwei Zhang, Zhenxing Liu, Qi Cai, Yu Xie, Yumin Liu, Li Peng

**Affiliations:** ^1^Department of Neurology, Zhongnan Hospital of Wuhan University, Wuhan, China; ^2^Department of Neurology, Yiling Hospital of Yichang, Yichang, China; ^3^Department of Cardiology, Zhongnan Hospital of Wuhan University, Wuhan, China

**Keywords:** ischemic stroke, albumin-to-alkaline phosphatase ratio, nonlinear relationship, unfavorable outcome, modified Rankin scale

## Abstract

**Background:**

The albumin-to-alkaline phosphatase ratio (AAPR) is a predictor of several disease outcomes. However, there is no study about AAPR and acute ischemic stroke outcomes. This study aims to investigate the relationship between AAPR and a 3-month unfavorable outcome in patients with acute ischemic stroke.

**Methods:**

This prospective cohort study included 2084 patients with acute ischemic stroke in South Korea. After applying strict exclusion criteria, 1,886 patients were included in our analysis and divided into three groups based on AAPR tertiles. An unfavorable outcome was defined as a 3-month modified Rankin scale (mRS) score > 2. Logistic regression analysis and smooth curve fitting analysis were applied to investigate the relationship between AAPR and unfavorable outcomes. Subgroup analysis was also performed to assess whether influencing factors changed the association between AAPR and unfavorable outcomes.

**Results:**

After adjusting for potential confounders, multivariate analysis showed that AAPR was significantly associated with a 3-month unfavorable outcome (OR 0.18, 95% CI 0.09–0.35, *p* < 0.001). The smooth curve fitting analysis showed a nonlinear relationship between AAPR and a 3-month unfavorable outcome. The infection point was 0.588 according to the recursive method, and the threshold analysis showed when AAPR was ≤0.588, with the per unit increase of AAPR, the 3-month unfavorable outcome risk decreased by 96% (OR 0.04, 95% CI 0.01–0.2, *p* < 0.001). However, when AAPR was >0.588, there was no negative correlation between AAPR and a 3-month unfavorable outcome (OR 0.33, 95% CI 0.08–1.3, *p* = 0.112).

**Conclusion:**

This study is the first to suggest a non-linear relationship between AAPR and a 3-month unfavorable outcome of acute ischemic stroke. AAPR was negatively correlated with a 3-month unfavorable outcome when AAPR was <0.588.

## Introduction

Acute ischemic stroke accounts for 80% of all stroke cases (1) and is one of the diseases that seriously threaten human life and health worldwide, with its high morbidity, mortality, disability, and recurrence rates (2, 3). In recent years, with the development of reperfusion therapy, especially the continuous progress of mechanical thrombectomy technology, the prognosis of acute ischemic stroke has been significantly improved; however, there are still a large number of patients with poor prognosis (4, 5). Accurate prediction of functional prognosis in patients with acute ischemic stroke can help make clinical decisions and help patients and their families conduct education and counseling (6). Therefore, a clinical index with high safety, repeatability, and simple convenience for predicting the prognosis of acute ischemic stroke is urgently needed in clinical practice. The 3-month modified Rankin scale (mRS) is widely used to evaluate the prognosis of acute ischemic stroke. An mRS score > 2 at 90 days is considered to indicate a poor prognosis (7, 8).

Studies have shown that hypoalbuminemia (9, 10) and elevated alkaline phosphatase (ALP) (11, 12) are risk factors for the poor prognosis of acute ischemic stroke. The albumin-to-alkaline phosphatase ratio (AAPR) is a new tumor prognostic indicator that can reflect systemic inflammation and nutritional status. It is easy to obtain, inexpensive, and superior to a single indicator (13, 14). However, studies on AAPR and the clinical prognosis of acute ischemic stroke have not been reported in the literature.

Therefore, we hypothesized that AAPR may still be a risk factor for poor prognosis in patients with acute ischemic stroke. In addition, considering the difference in AAPR ratio distribution, there may be a nonlinear relationship between the AAPR ratio and poor prognosis in patients with acute ischemic stroke. This study investigated the linear or non-linear relationship between AAPR and poor prognosis by conducting a secondary analysis of a cohort study in Korea.

## Methods

### Data source

The raw data was obtained from a prospective cohort study in South Korea (15), and it was an open-access article distributed under the terms of the Creative Commons Attribution License, permitting unrestricted use and reproduction.

### Study population

The data from a prospective cohort study in South Korea recruited 2,084 patients with acute ischemic stroke admitted within the first 7 days of stroke onset from January 2010 to December 2016. This study was approved by Seoul National University Hospital’s Institutional Review Board (Approval No. 1009-062-332), and the consent of the patients was waived. For the secondary analysis, no additional ethical approval was required. Strict exclusion criteria were applied to this study for cases lacking dysphagia test results, relevant laboratory examination upon admission, and records of the mRS 3 months after acute ischemic stroke. An unfavorable outcome was defined as a 3-month mRS score > 2, and a favorable outcome was defined as a 3-month mRS 0–2.

### Variables

Variables, including continuous and categorical variables, were collected from the raw data file. Continuous variables were body mass index (BMI), white blood cells (WBC), hemoglobin, hematocrit (HCT), mean corpuscular volume (MCV), mean corpuscular hemoglobin (MCH), mean corpuscular hemoglobin concentration (MCHC), red cell distribution width (RDW), platelets, total serum cholesterol (TC), triglyceride (TG), serum high-density lipoprotein cholesterol (HDL-C), serum low-density lipoproteins cholesterol (LDL-C), blood urea nitrogen (BUN), creatinine, alanine aminotransferase (ALT), aspartate aminotransferase (AST), alkaline phosphatase (ALP), albumin, protein, albumin-to-alkaline phosphatase ratio (AAPR), National Institute of Health Stroke Scale (NIHSS) on admission, and 3-month modified Rankin scale (mRS). Categorical variables were age, sex, previous stroke/ Transient Ischemic Attack (TIA), hypertension, diabetes mellitus, smoking, atrial fibrillation, coronary heart disease, and ischemic stroke etiology. AAPR was considered a continuous variable and transformed into a categorical variable based on the following tertiles: T1 (<0.507), T2 (0.507–0.666), and T3 (>0.666).

### Missing data processing

There were missing data for several variables in our study: HDL-C had 99 cases (5.19%), LDL-C had 75 cases (3.93%), TG had 107 cases (5.61%), and TC had 1 case (0.00%). Multiple imputations were applied to address these missing values and mitigate bias and uncertainty during the modeling process.

### Statistical analysis

SPSS 29 (Statistical Package for the Social Sciences) and R software (The R Foundation^1^) were used for data processing. Continuous variables were represented by mean ± standard deviation or median (interquartile ranges), and categorical variables as frequencies and percentages. Comparisons were made using analysis of variance (ANOVA) for distributed data, or the Kruskal-Wallis test for skewed data, and the χ^2^ test for categorical data. Logistic regression analysis was performed to investigate the association between AAPR and a 3-month unfavorable outcome. We used three models to improve the precision of the results. Model 1 is adjusted for age and sex; Model 2 is adjusted for age, sex, BMI, previous stroke/TIA, hypertension, diabetes mellitus, hyperlipidemia, smoking, atrial fibrillation, coronary heart disease, ischemic stroke etiology, and NIHSS on admission; Model 3 is further adjusted for age, sex, BMI, previous stroke/TIA, hypertension, diabetes mellitus, hyperlipidemia, smoking, atrial fibrillation, coronary heart disease, ischemic stroke etiology, NIHSS on admission, TC, TG, HDL-C, LDL-C, BUN, creatinine, ALT, and AST. To investigate the potential nonlinear relationship between AAPR and a 3-month unfavorable outcome, a smooth curve fitting analysis by GAM (Generalized Additive Model) was formulated, and the adjusted variables for the analysis were based on variables for Model 3. Finally, we conducted a subgroup analysis to assess the robustness of the relationship between AAPR and a 3-month unfavorable outcome, including age, sex, BMI, previous stroke/TIA, diabetes mellitus, hypertension, hyperlipidemia, smoking, atrial fibrillation, coronary heart disease, NIHSS on admission, and ischemic stroke etiology. The *p* < 0.05 was considered statistically significant.

## Results

### Baseline characteristics of the patients

**Figure 1:**
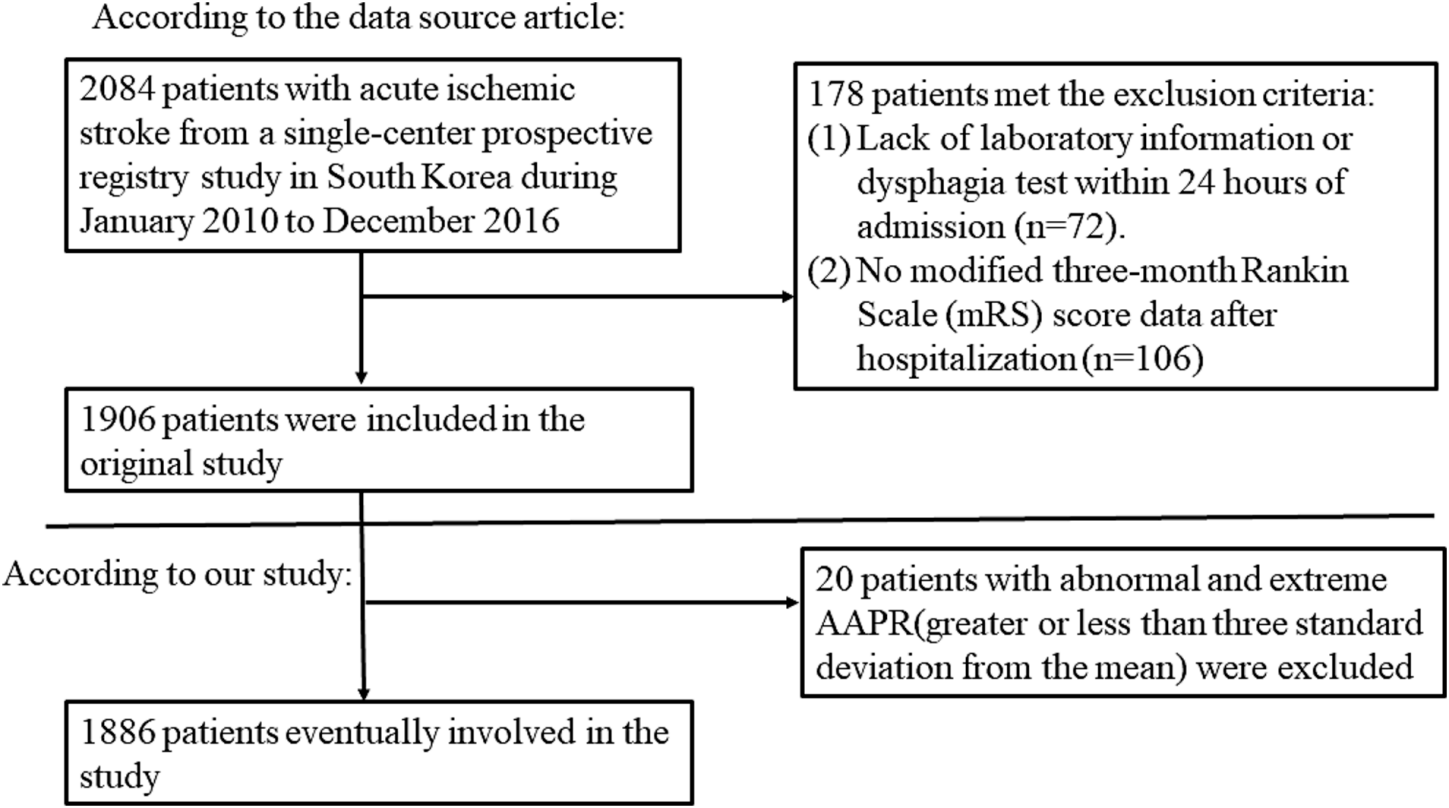
Flowchart of patient selection and exclusion.

After applying strict inclusion and exclusion criteria, 1886 patients were included in our analysis, and the flowchart is shown in Figure 1. There were 1,157 (61.35%) females and 729 (38.65%) males. Of the total patients, 432 (22.91%) were < 60 years and 293 (15.54%) were > 80 years. There were 1,202 (63.73%) patients with hypertension and 610 (32.34%) patients with diabetes mellitus. The etiology of ischemic stroke showed that 605 (32.08%) cases were large-artery atherosclerosis (LAA), 357 (18.93%) cases were small vessel occlusion (SVO), 484 (25.66%) cases were cardiogenic embolism (CE), 171 (9.07%) cases were other determined, and 269 (14.26%) cases were undetermined. The baseline characteristics based on tertiles of AAPR are presented in Table 1. Compared to the first tertile, there were more patients with favorable outcomes, more patients under 60 years old, more females, higher RBC levels, higher levels of hemoglobin, albumin, HCT, MCH, MCHC, HDL-C, and protein, and more patients with ischemic stroke type of LAA in the third tertile.

**Table 1:** Baseline characteristics of the patients.

Variables	Total *n* = 1,886	AAPR T1 (<0.507) *n* = 629	AAPR T2 (0.507–0.666) *n* = 622	AAPR T3 (>0.666) *n* = 635	*P* value
Outcome, *n* (%)					<0.001
Favorable outcome	1,342 (71.16%)	376 (59.78%)	456 (73.31%)	510 (80.31%)	
Unfavorable outcome	544 (28.84%)	253 (40.22%)	166 (26.69%)	125 (19.69%)	
Age, years, *n* (%)					<0.001
<60	432 (22.91%)	107 (17.01%)	136 (21.86%)	189 (29.76%)	
≥80	293 (15.54%)	132 (20.99%)	92 (14.79%)	69 (10.87%)	
60–70	497 (26.35%)	159 (25.28%)	167 (26.85%)	171 (26.93%)	
70–80	664 (35.21%)	231 (36.72%)	227 (36.50%)	206 (32.44%)	
Sex, *n* (%)					0.001
Female	1,157 (61.35%)	349 (55.48%)	400 (64.31%)	408 (64.25%)	
Male	729 (38.65%)	280 (44.52%)	222 (35.69%)	227 (35.75%)	
WBC, 10^9^/L	8.14 (2.90)	8.39 (3.29)	8.11 (2.71)	7.92 (2.63)	0.014
RBC, 10^9^/L	4.32 (0.64)	4.19 (0.68)	4.39 (0.64)	4.39 (0.59)	<0.001
Hemoglobin	13.48 (2.00)	12.99 (2.12)	13.71 (1.96)	13.73 (1.83)	<0.001
HCT, %	40.07 (5.60)	38.77 (5.96)	40.76 (5.47)	40.69 (5.12)	<0.001
MCV, fL	92.94 (5.19)	92.75 (5.53)	93.17 (5.12)	92.91 (4.89)	0.346
MCH, g/dL	31.23 (1.99)	31.05 (2.18)	31.31 (1.87)	31.33 (1.89)	0.023
MCHC, g/dL	33.60 (1.14)	33.47 (1.17)	33.61 (1.12)	33.72 (1.10)	0.001
RDW, %	13.39 (1.52)	13.79 (1.73)	13.29 (1.45)	13.10 (1.25)	<0.001
Platelet, 10^9^/L	223.20 (70.72)	227.25 (81.47)	222.77 (63.11)	219.60 (66.06)	0.155
TC, mg/dL	179.29 (44.06)	177.47 (46.60)	181.95 (44.26)	178.48 (41.13)	0.169
TG, mg/dL	111.21 (56.57)	111.25 (57.73)	110.57 (53.26)	111.78 (58.59)	0.930
HDL-c, mg/dL	46.59 (13.67)	45.41 (14.29)	46.75 (13.02)	47.61 (13.58)	0.016
LDL-c, mg/dL	108.13 (37.65)	108.24 (38.49)	111.01 (38.44)	105.19 (35.81)	0.023
BUN, mg/dL	17.61 (8.91)	18.78 (10.50)	16.73 (8.06)	17.32 (7.82)	<0.001
Creatinine, mg/dL	1.09 (1.04)	1.20 (1.29)	1.02 (0.90)	1.04 (0.87)	0.003
AST, U/L	26.12 (14.39)	29.36 (19.91)	24.71 (11.05)	24.30 (9.39)	<0.001
ALT, U/L	22.42 (16.14)	24.11 (20.17)	21.54 (14.03)	21.60 (13.21)	0.005
ALP, U/L	77.22 (48.38)	110.34 (71.29)	69.73 (7.91)	51.76 (8.46)	<0.001
Albumin, g/L	40.17 (4.28)	38.19 (4.90)	40.67 (3.59)	41.63 (3.44)	<0.001
Protein, g/L	7.01 (0.61)	6.92 (0.70)	7.01 (0.55)	7.09 (0.57)	<0.001
BMI, kg/m^2^	23.48 (3.26)	23.01 (3.25)	23.51 (3.34)	23.92 (3.12)	<0.001
Previous stroke/TIA, *n* (%)					0.095
No	1,489 (78.95%)	480 (76.31%)	493 (79.26%)	516 (81.26%)	
Yes	397 (21.05%)	149 (23.69%)	129 (20.74%)	119 (18.74%)	
Hypertension, *n* (%)					0.027
No	684 (36.27%)	206 (32.75%)	249 (40.03%)	229 (36.06%)	
Yes	1,202 (63.73%)	423 (67.25%)	373 (59.97%)	406 (63.94%)	
Diabetes mellitus, *n* (%)					0.629
No	1,276 (67.66%)	418 (66.45%)	420 (67.52%)	438 (68.98%)	
Yes	610 (32.34%)	211 (33.55%)	202 (32.48%)	197 (31.02%)	
Hyperlipidemia, *n* (%)					0.581
No	1,192 (63.20%)	406 (64.55%)	394 (63.34%)	392 (61.73%)	
Yes	694 (36.80%)	223 (35.45%)	228 (36.66%)	243 (38.27%)	
Smoking, *n* (%)					0.008
No	1,405 (74.50%)	486 (77.27%)	436 (70.10%)	483 (76.06%)	
Yes	481 (25.50%)	143 (22.73%)	186 (29.90%)	152 (23.94%)	
Atrial fibrillation, *n* (%)					0.017
No	1,486 (78.79%)	472 (75.04%)	505 (81.19%)	509 (80.16%)	
Yes	400 (21.21%)	157 (24.96%)	117 (18.81%)	126 (19.84%)	
Coronary heart disease, *n* (%)					0.012
No	1,667 (88.39%)	571 (90.78%)	553 (88.91%)	543 (85.51%)	
Yes	219 (11.61%)	58 (9.22%)	69 (11.09%)	92 (14.49%)	
NIHSS upon admission	5.41 (5.74)	6.59 (6.19)	5.19 (5.48)	4.46 (5.32)	<0.001
Ischemic stroke etiology, *n* (%)					<0.001
LAA	605 (32.08%)	168 (26.71%)	227 (36.50%)	210 (33.07%)	
SVO	357 (18.93%)	110 (17.49%)	122 (19.61%)	125 (19.69%)	
CE	484 (25.66%)	174 (27.66%)	142 (22.83%)	168 (26.46%)	
Other determined	171 (9.07%)	84 (13.35%)	42 (6.75%)	45 (7.09%)	
Undetermined	269 (14.26%)	93 (14.79%)	89 (14.31%)	87 (13.70%)	
NIHSS upon admission					<0.001
<6	1,282 (67.97%)	365 (58.03%)	428 (68.81%)	489 (77.01%)	
≥13	250 (13.26%)	119 (18.92%)	70 (11.25%)	61 (9.61%)	
6–12	354 (18.77%)	145 (23.05%)	124 (19.94%)	85 (13.39%)	
3-month mRS	1.77 (1.79)	2.31 (2.03)	1.66 (1.70)	1.35 (1.46)	<0.001
AAPR	0.60 (0.20)	0.39 (0.10)	0.59 (0.05)	0.82 (0.13)	0.000

BMI, body mass index; WBC, white blood cells; HCT, hematocrit; MCV, corpuscular volume; MCH, corpuscular hemoglobin; MCHC, corpuscular hemoglobin concentration; RDW, red cell distribution width; TC, total serum cholesterol; TG, triglyceride; HDL-C, high-density lipoprotein cholesterol; LDL-C, low-density lipoproteins cholesterol; BUN, blood urea nitrogen; ALT, alanine aminotransferase; AST, aspartate aminotransferase; ALP, alkaline phosphatase; AAPR, albumin-to-alkaline phosphatase ratio; NIHSS, National Institute of Health Stroke Scale; mRS, modified Rankin scale; SVO, small vessel occlusion; LAA, large-artery atherosclerosis; CE, cardiogenic embolism.

### Univariate analysis

The univariate analysis (Table 2) showed a negative correlation between AAPR and a 3-month unfavorable outcome (OR 0.07, 95% CI 0.04–0.12, *p* < 0.001). Meanwhile, the univariate logistic analysis also showed that male sex, WBC count, RDW, BUN, creatinine, ALT, AST, ALP, previous stroke/TIA, hypertension, diabetes mellitus, atrial fibrillation, and NIHSS on admission were positively associated with a 3-month unfavorable outcome (*p* < 0.05). However, RBC, hemoglobin, HCT, MCH, MCHC, ALT, albumin, protein, BMI, hyperlipidemia, and smoking were negatively correlated with a 3-month unfavorable outcome (*p* < 0.05).

**Table 2:** Univariate analysis.

Variables	OR (95% CI)	*P* value
Age, years, *n* (%)		
<60	Ref.	Ref.
60–70	1.15 (0.84–1.59)	0.393
70–80	1.90 (1.43–2.55)	<0.001
≥80	3.94 (2.84–5.50)	0.000
Sex, *n* (%)		
Female	Ref.	Ref.
Male	1.65 (1.35–2.02)	<0.001
WBC, 10^9^/L	1.08 (1.04–1.12)	<0.001
RBC, 10^9^/L	0.58 (0.50–0.68)	<0.001
Hemoglobin, g/dL	0.82 (0.78–0.86)	<0.001
HCT, %	0.93 (0.92–0.95)	<0.001
MCV, fL	0.99 (0.97–1.01)	0.209
MCH, g/dL	0.93 (0.89–0.98)	0.005
MCHC, g/dL	0.86 (0.79–0.94)	0.001
RDW, %	1.25 (1.17–1.33)	<0.001
Platelet, 10^9^/L	1.00 (1.00–1.00)	0.253
TC, mg/dL	1.00 (0.99–1.00)	<0.001
TG, mg/dL	1.00 (0.99–1.00)	0.001
HDL-C, mg/dL	1.00 (0.99–1.01)	0.570
LDL-C, mg/dL	1.00 (0.99–1.00)	0.021
BUN, mg/dL	1.02 (1.01–1.03)	0.003
Creatinine, mg/dL	1.02 (0.93–1.12)	0.737
AST, U/L	1.01 (1.00–1.02)	0.008
ALT, U/L	0.99 (0.99–1.00)	0.043
ALP, U/L	1.01 (1.01–1.01)	<0.001
Albumin, g/L	0.88 (0.86–0.90)	<0.001
Protein, g/L	0.68 (0.58–0.80)	<0.001
BMI, kg/m^2^	0.92 (0.89–0.95)	<0.001
Previous stroke/TIA		
No	Ref.	Ref.
Yes	1.81 (1.43–2.28)	<0.001
Hypertension, *n* (%)		
No	Ref.	Ref.
Yes	1.32 (1.07–1.63)	0.010
Diabetes mellitus, *n* (%)		
No	Ref.	Ref.
Yes	1.43 (1.16–1.76)	0.001
Hyperlipidemia, *n* (%)		
No	Ref.	Ref.
Yes	0.78 [0.63;0.96]	0.019
Smoking, *n* (%)		
No	Ref.	Ref.
Yes	0.78 (0.62–0.99)	0.038
Atrial fibrillation, *n* (%)		
No	Ref.	Ref.
Yes	2.02 (1.60–2.55)	<0.001
Coronary heart disease, *n* (%)		
No	Ref.	Ref.
Yes	1.02 (0.75–1.39)	0.889
NIHSS upon admission	1.23 (1.20–1.25)	<0.001
Ischemic stroke etiology		
LAA	Ref.	Ref.
SVO	0.63 (0.45–0.86)	0.004
CE	1.52 (1.17–1.97)	0.001
Other determined	2.07 (1.45–2.94)	<0.001
Undetermined	0.88 (0.63–1.22)	0.458
NIHSS upon admission		
<6	Ref.	Ref.
6–12	6.34 (4.89–8.24)	0.000
≥13	15.1 (11.0–20.8)	0.000
AAPR	0.07 (0.04–0.12)	<0.001

BMI, body mass index; WBC, white blood cells; HCT, hematocrit; MCV, corpuscular volume; MCH, corpuscular hemoglobin; MCHC, corpuscular hemoglobin concentration; RDW, red cell distribution width; TC, total serum cholesterol; TG, triglyceride; HDL-C, high-density lipoprotein cholesterol; LDL-C, low-density lipoproteins cholesterol; BUN, blood urea nitrogen; ALT, alanine aminotransferase; AST, aspartate aminotransferase; ALP, alkaline phosphatase; AAPR, albumin-to-alkaline phosphatase ratio; NIHSS, National Institute of Health Stroke Scale; mRS, modified Rankin scale; SVO, small vessel occlusion; LAA, large-artery atherosclerosis; CE, cardiogenic embolism.

### Univariate and multivariate logistic regression analyses

Multivariate logistic regression analysis was performed to explore the association between AAPR and a 3-month unfavorable outcome. The non-adjusted model showed a 93% reduction in the risk of an unfavorable outcome for each unit increase in the AAPR [OR 0.07, 95% CI (0.04–0.12), *p* < 0.001] (Table 3). Models 1, 2, and 3 revealed a 91, 80, and 82% reduction in the risk of developing an unfavorable outcome for each unit increase in the AAPR [OR 0.09, 95%CI (0.05–0.16); OR 0.2, 95%CI (0.1–0.38); OR 0.18, 95%CI (0.09–0.35)], respectively, showing statistical significance. When AAPR was converted to a categorical variable, the groups with higher AAPR were less likely to develop an unfavorable outcome than the T1 group [T2 group: OR 0.54, 95%CI (0.43–0.69), *p* < 0.001; T3 group: OR 0.36, 95%CI (0.28–0.47), *p* < 0.001] and the risk of developing an unfavorable outcome was decreased in patients with a higher AAPR in Model 3 [T2 group: OR 0.7, 95%CI (0.53–0.94), *p* = 0.016; T3 group: OR 0.56, 95%CI (0.41–0.76), *p* < 0.001] (Table 3). There were no statistically significant differences in the trends (*p* < 0.001).

**Table 3:** Univariate and multivariate logistic regression analyses.

	Non-adjusted		Model 1		Model 2		Model3	
Variable	OR (95% CI)	*P*	OR (95% CI)	*P*	OR (95% CI)	*P*	OR (95% CI)	*P*
AAPR	0.07 (0.04–0.12)	<0.001	0.09 (0.05–0.16)	<0.001	0.2 (0.1–0.38)	<0.001	0.18 (0.09–0.35)	<0.001
Trisections of AAPR								
T1 (<0.507)	Ref.		Ref.		Ref.		Ref.	
T2 (0.507–0.666)	0.54 (0.43–0.69)	<0.001	0.58 (0.46–0.74)	<0.001	0.71 (0.53–0.94)	<0.001	0.7 (0.53–0.94)	0.016
T3 (>0.666)	0.36 (0.28–0.47)	<0.001	0.42 (0.32–0.54)	<0.001	0.57 (0.42–0.77)	<0.001	0.56 (0.41–0.76)	<0.001
P for trend	0.6 (0.53–0.68)	<0.001	0.64 (0.56–0.73)	<0.001	0.75 (0.65–0.87)	<0.001	0.75 (0.64–0.87)	<0.001

Model 1 is adjusted for age and sex.

Model 2 is adjusted for age, sex, BMI, previous stroke/TIA, hypertension, diabetes mellitus, hyperlipidemia, smoking, atrial fibrillation, coronary heart disease, ischemic stroke etiology, and NIHSS on admission.

Model 3 is adjusted for age, sex, BMI, previous stroke/TIA, hypertension, diabetes mellitus, hyperlipidemia, smoking, atrial fibrillation, coronary heart disease, ischemic stroke etiology, NIHSS on admission, TC, TG, HDL-C, LDL-C, BUN, creatinine, ALT, and AST.

### Nonlinear relationship between AAPR and a 3-month unfavorable outcome

**Figure 2:**
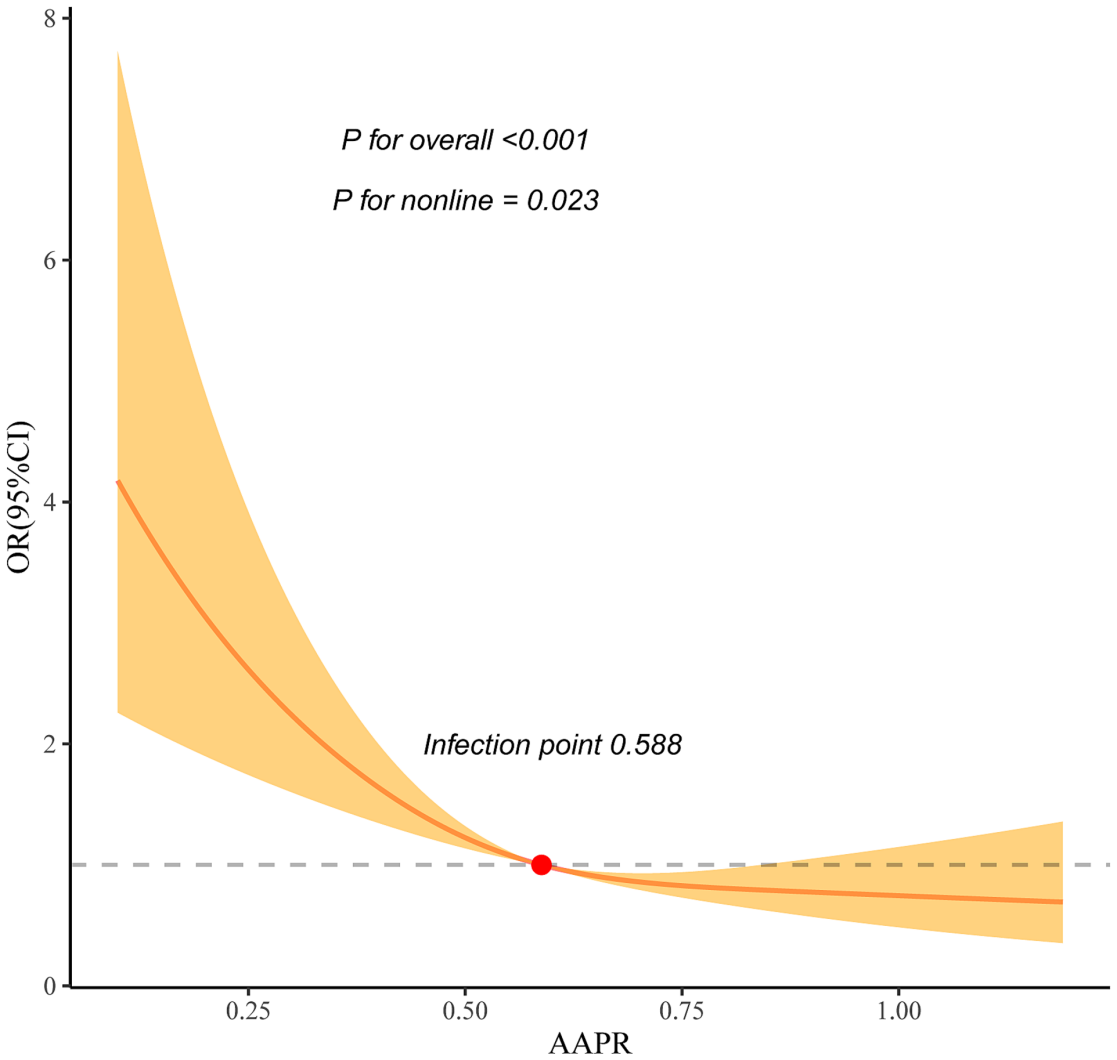
Nonlinear relationship between AAPR and a 3-month unfavorable outcome. Smooth-fitting curve analysis showed a nonlinear relationship between AAPR and a 3-month unfavorable outcome (p for nonlinear = 0.023) after adjusting for age, sex, BMI, previous stroke/TIA, hypertension, diabetes mellitus, hyperlipidemia, smoking, atrial fibrillation, coronary heart disease, ischemic stroke etiology, NIHSS on admission, TC, TG, HDL-C, LDL-C, BUN, creatinine, ALT, and AST.

The smooth curve fitting analysis showed an L-shaped relationship between AAPR and the risk of a 3-month unfavorable outcome (p for nonlinear = 0.023) after we adjusted covariates in Model 3 (Figure 2). The infection point was 0.588 according to the recursive method, and the threshold analysis showed when AAPR ≤0.588, with the per-unit increase in AAPR, the 3-month unfavorable outcome risk decreased by 96% (OR 0.04, 95% CI 0.01–0.2, *p* < 0.001). However, when AAPR was >0.588, there was no negative correlation between AAPR and a 3-month unfavorable outcome (OR 0.33, 95% CI 0.08–1.3, *p* = 0.112) (Table 4).

**Table 4:** The results of the two-piecewise regression model.

Unfavorable outcome	OR (95%CI)	*P*
Fitting the model using standard linear regression	0.18 (0.09–0.35)	<0.001
Fitting the model using two-piece linear regression		
Inflection point	0.588	
<0.588	0.04 (0.01–0.2)	<0.001
>0.588	0.33 (0.08–1.3)	0.112
P for log-likelihood ratio test		<0.001

### Subgroup analysis

In the subgroup analysis (Table 5), age, sex, BMI, previous stroke/TIA, diabetes mellitus, hypertension, hyperlipidemia, smoking, atrial fibrillation, coronary heart disease, and NIHSS on admission all showed no significant interaction with AAPR (p for interaction > 0.05). However, a significant interaction was observed between ischemic stroke etiology and the AAPR (p for interaction = 0.044).

**Table 5:** Subgroup analysis of the relationship between AAPR and a 3-month function outcome.

	Total *N* = 1,886	Favorable outcome *N* = 1,342	Unfavorable outcome *N* = 544	OR (95%CI)	*P*	P for interaction
Sex, *n* (%)						0.834
Female	1,157 (61.35%)	870 (64.83%)	287 (52.76%)	0.2 (0.08–0.47)	0	
Male	729 (38.65%)	472 (35.17%)	257 (47.24%)	0.14 (0.05–0.38)	0	
Age, years, *n* (%)						0.772
<70	929 (49.26%)	737 (54.92%)	192 (35.29%)	0.16 (0.06–0.43)	0	
≥70	957 (50.74%)	605 (45.08%)	352 (64.71%)	0.19 (0.08–0.44)	0	
BMI, kg/m2						0.217
≤23.4	939 (49.79%)	626 (46.65%)	313 (57.54%)	0.1 (0.04–0.26)	0	
>23.4	947 (50.21%)	716 (53.35%)	231 (42.46%)	0.25 (0.1–0.66)	0.005	
Previous stroke/TIA, *n* (%)						0.234
No	1,489 (78.95%)	1,100 (81.97%)	389 (71.51%)	0.24 (0.12–0.51)	0	
Yes	397 (21.05%)	242 (18.03%)	155 (28.49%)	0.04 (0.01–0.17)	0	
Hypertension, *n* (%)						0.353
No	684 (36.27%)	511 (38.08%)	173 (31.80%)	0.25 (0.08–0.76)	0.015	
Yes	1,202 (63.73%)	831 (61.92%)	371 (68.20%)	0.14 (0.06–0.31)	0	
Diabetes mellitus, *n* (%)						0.445
No	1,276 (67.66%)	939 (69.97%)	337 (61.95%)	0.16 (0.07–0.36)	0	
Yes	610 (32.34%)	403 (30.03%)	207 (38.05%)	0.26 (0.08–0.8)	0.019	
Hyperlipidemia, *n* (%)						0.325
No	1,192 (63.20%)	826 (61.55%)	366 (67.28%)	0.14 (0.06–0.33)	0	
Yes	694 (36.80%)	516 (38.45%)	178 (32.72%)	0.26 (0.09–0.79)	0.018	
Smoking, *n* (%)						0.975
No	1,405 (74.50%)	982 (73.17%)	423 (77.76%)	0.18 (0.08–0.36)	0	
Yes	481 (25.50%)	360 (26.83%)	121 (22.24%)	0.15 (0.03–0.65)	0.011	
Atrial fibrillation, *n* (%)						0.511
No	1,486 (78.79%)	1,106 (82.41%)	380 (69.85%)	0.15 (0.07–0.31)	0	
Yes	400 (21.21%)	236 (17.59%)	164 (30.15%)	0.29 (0.08–1.13)	0.074	
Coronary heart disease, *n* (%)						0.615
No	1,667 (88.39%)	1,187 (88.45%)	480 (88.24%)	0.18 (0.09–0.37)	0	
Yes	219 (11.61%)	155 (11.55%)	64 (11.76%)	0.08 (0.01–0.63)	0.017	
NIHSS on admission						0.917
<6	1,282 (67.97%)	1,099 (81.89%)	183 (33.64%)	0.15 (0.06–0.39)	0	
≥6	604 (32.03%)	243 (18.11%)	361 (66.36%)	0.17 (0.06–0.43)	0	
Ischemic stroke etiology, *n* (%)						0.044
LAA	605 (32.08%)	442 (32.94%)	163 (29.96%)	0.18(0.05–0.62)	0.006	
SVO	357 (18.93%)	290 (21.61%)	67 (12.32%)	1.05(0.18–6.24)	0.955	
CE	484 (25.66%)	310 (23.10%)	174 (31.99%)	0.19(0.06–0.62)	0.006	
Other determined	171 (9.07%)	97 (7.23%)	74 (13.60%)	0.02(0–0.18)	0.001	
Undetermined	269 (14.26%)	203 (15.13%)	66 (12.13%)	0.06(0–0.64)	0.02	

## Discussion

Our results demonstrated for the first time that AAPR was an independent risk factor for a 3-month unfavorable outcome of acute ischemic stroke. The two-piecewise regression model suggested that AAPR was nonlinearly correlated with an unfavorable outcome. The threshold analysis showed when AAPR ≤0.588, with the per unit increase in AAPR, the 3-month unfavorable outcome risk decreased by 96% (OR 0.04, 95% CI 0.01–0.2, *p* < 0.001). However, when AAPR was >0.588, there was no negative correlation between AAPR and a 3-month unfavorable outcome (OR 0.33, 95% CI 0.08–1.3, *p* = 0.112).

Albumin is a multifunctional protein with the highest proportion in human plasma. It is synthesized and secreted into the blood by liver cells and has many physiological properties, including binding with a variety of endogenous and exogenous substances (such as inorganic ions, fatty acids, bilirubin, vitamins, hormones, steroids, and drugs), antioxidant, anticoagulant, and other effects (16). Albumin is an important inflammatory regulator and free radical scavenger in the body (17). In patients with acute ischemic stroke, albumin may improve the prognosis of stroke by improving cerebral blood perfusion, enhancing microvascular perfusion, reducing the adhesion of various cytokines in the microcirculation in capillaries, and increasing the transport of free fatty acids after stroke (18). The prevalence of low serum albumin in ischemic stroke was 20–25% (19), which was not only related to the patient’s previous nutritional status but also may be related to the increased protein consumption caused by post-stroke stress reaction, co-infection, and other complications, and the insufficient protein intake caused by secondary swallowing or limb dysfunction. Previous studies have found that hypoalbuminemia is significantly associated with poor prognosis in patients with acute ischemic stroke (20, 21). Babu et al. (22) conducted a prospective study with 560 patients with ischemic stroke and found that low albumin levels were significantly associated with poor prognosis (OR 1.972, 95% CI 1.103–4.001), defined by the mRS score > 3 at 3 months after stroke, and the stroke recurrence rate was higher in patients with low albumin levels compared with those with high albumin levels. Additionally, they also found that low albumin levels were significantly associated with poor prognosis in all subtypes of stroke etiology according to TOAST (Trial of ORG 10172 in Acute Stroke Treatment). In a prospective study with 444 patients with ischemic stroke conducted by Idicula et al. (23), they found that high serum albumin levels were independently related to a better prognosis (OR 1.12, 95% CI 1.05–1.20), and the mortality was lower in patients with high albumin levels compared with those with low albumin levels by Cox regression analysis adjusted for age, sex, and NIHSS score on admission (OR 0.88, 95% CI 0.83–0.93). In addition, some studies have shown that serum albumin has a protective effect on nerve cells, but the mechanism remains unclear. Baltanas et al. (24) found that adding bovine plasma albumin to cultured cerebral cortical nerve cells *in vitro* could reduce their DNA damage and apoptosis rate, suggesting that the neuroprotective effect of albumin may be related to its antioxidant mechanism. Bento-Abreu et al. (25) found that albumin could affect the metabolism of astrocytes cultured *in vitro*, entering into astrocytes through pinocytosis and stimulating the synthesis of oleic acid, a neurotrophic factor. Ralay Ranaivo et al. (26) found that albumin could activate astrocytes and microglia and induce cellular damage and repair mechanisms through the mitogen-activated protein kinase pathway.

ALP is a metalloenzyme in membrane-bound glycoprotein and an enzyme that catalyzes calcification inhibitor pyrophosphate hydrolysis. ALP is widely distributed in various organs and tissues of the human body, with the highest activity observed in the liver. Abnormal ALP levels were observed in hepatobiliary diseases, such as liver cancer and obstructive jaundice, as well as intestinal diseases, metabolic abnormalities, chronic renal insufficiency, and bone metabolism abnormalities (27, 28). Studies have shown that ALP was a new inflammatory index in cardiovascular and cerebrovascular diseases, and the increase in serum ALP level was associated with a variety of atherosclerotic diseases, suggesting that ALP may be a risk factor for cardiovascular and cerebrovascular diseases (29–31). Kim et al. (31) conducted a retrospective observational study with 1,034 patients admitted with a first-ever acute ischemic stroke, and their results suggested that higher ALP levels were significantly associated with poor prognosis (OR 1.25; 95% CI 1.04–1.50), defined by the mRS score > 2 at 3 months after stroke. In a multicenter retrospective study with 2,944 patients with ischemic stroke, Zhong et al. (32) found a linear relationship between ALP and in-hospital mortality (p for linearity = 0.017), and the multivariable logistic regression model showed the hazard ratio was 2.19 (95% CI 1.20–4.00) for in-hospital mortality in the highest quartile of ALP compared with the lowest quartile, suggesting that ALP levels were associated with poor neurological recovery, increased mortality, and poor overall prognosis in patients with ischemic stroke. A possible mechanism by which a high ALP level leads to a poor prognosis of ischemic stroke is that ALP can deactivate mechanical pyrophosphate through hydrolysis and accelerate vascular calcification (33, 34). ALP can also mediate vascular calcification by inducing venous collagen deposition in the blood vessel wall, leading to microvascular dysfunction (35, 36). Vascular calcification leads to vascular hardening, degeneration, and atherosclerosis (37). With the increase in serum ALP levels, vascular calcification was obvious, which promoted the occurrence of vascular disease events; ALP was highly expressed in cerebral microvascular endothelial cells, which were particularly important in maintaining vascular homeostasis (38, 39); elevated serum ALP was found to be associated with vascular endothelial cell dysfunction in patients with essential hypertension (40).

AAPR is a simple and low-cost new indicator calculated from routine biochemical examination. It is often used to evaluate systemic inflammation and the nutritional status of the body (41). At present, low levels of AAPR have been found to be significantly associated with poor prognosis in patients with various malignant tumors, such as renal cell carcinoma, lung cancer, breast cancer, and cholangiocarcinoma (42–45). AAPR provided clinicians with more information than a single indicator of reduced albumin or elevated ALP and could help identify more patients with poor clinical outcomes (46). A retrospective cohort study showed that AAPR was nonlinearly correlated with the prognosis of small-cell lung cancer (42). Similar to our findings, we also found that AAPR was non-linearly correlated with the prognosis of acute ischemic stroke.

In the present study, AAPR, as a composite indicator, may reflect the balance between inflammation and nutritional status in patients. A low AAPR value indicates an inflammatory response that dominates the patient’s body, accompanied by poor nutritional status, which may lead to adverse clinical outcomes. Conversely, a high AAPR value may suggest that the patient’s nutritional status is relatively good, enabling them to better resist inflammatory responses, thereby contributing to improved prognosis. However, our study also found that when the AAPR exceeds a certain threshold, its correlation with prognosis becomes insignificant. This may be because at high AAPR levels, other factors begin to exert a more dominant influence on prognosis, or the predictive value of AAPR is most significant within a specific range. These findings emphasize the importance of considering the nonlinear characteristics of AAPR in clinical practice and suggest that physicians should comprehensively consider AAPR values along with other clinical and laboratory indicators when assessing patients with acute ischemic stroke. Future research should further explore the specific mechanisms and application value of AAPR in the prognostic assessment of acute ischemic stroke.

Integration into existing risk scores could involve incorporating AAPR as an additional variable in the scoring system, potentially enhancing its predictive accuracy. For instance, in the THRIVE (Transnasal Humidified Rapid-Insufflation Ventilatory Exchange) (47) and SPAN-100 [Stroke Prognostication using Age and National Institute of Health Stroke Scale (NIHSS)] scores (48), incorporating AAPR might help better stratify patients into different risk categories. Guiding intervention measures involve the use of AAPR as a basis for tailoring treatment plans. For example, patients with lower AAPR values may benefit from more aggressive nutritional support to improve their albumin levels and potentially their overall prognosis. Conversely, patients with higher AAPR values may require different types of interventions tailored to their specific needs. Overall, further research is needed to explore the potential clinical utility of AAPR and to determine how it can be best integrated into clinical practice to improve patient outcomes.

However, our study has some limitations. First, the single-center retrospective study, mainly involving the Korean population, had clinical selection bias, and the results of this study needed to be further validated in a multi-center, large-sample, diverse prospective cohort study. Second, AAPR was assessed on admission, and there were no data during treatment. Thus, further research on the dynamic evolution of AAPR is needed. Third, this study was a secondary analysis, and we did not know whether the specific treatment of the patients in this study, such as standard medical treatment, thrombolysis, or mechanical thrombectomy, may have influenced the results. The absence of these data points introduces complexity and potential bias. For instance, patients who undergo thrombolysis or thrombectomy may experience different clinical trajectories compared with those who do not, and this treatment variability could impact the observed relationship between AAPR and prognosis. Future studies should aim to include comprehensive treatment data to better understand the interplay between AAPR and treatment interventions. Finally, some variables in this raw data were incomplete. For example, stratifying ages by 10 years, but not specific ages, potentially leads to incomplete data on certain variable information. Further information on specific clinical variables is required to be collected in the future.

## Conclusion

Our study is the first to suggest a non-linear relationship between AAPR and a 3-month unfavorable outcome of acute ischemic stroke. AAPR was negatively correlated with a 3-month unfavorable outcome when AAPR was <0.588. Our findings may help guide treatment decisions and identify potential interventions to further improve favorable outcomes for patients with acute ischemic stroke.

## Data Availability

Publicly available datasets were analyzed in this study. This data can be found here: https://doi.org/10.1371/journal.pone.0228738.s001.

## Footnotes

^1^ http://www.R-project.org
